# The PACE Study: A randomised clinical trial of cognitive activity (CA) for older adults with mild cognitive impairment (MCI)

**DOI:** 10.1186/1745-6215-10-114

**Published:** 2009-12-14

**Authors:** Mandy R Vidovich, Nicola T Lautenschlager, Leon Flicker, Linda Clare, Osvaldo P Almeida

**Affiliations:** 1 Western Australia Centre for Health and Ageing, School of Psychiatry and Clinical Neurosciences, Perth, Western Australia, Australia; 2 School of Psychiatry and Clinical Neurosciences, University of Western Australia, Perth, Western Australia, Australia; 3 School of Medicine and Pharmacology, University of Western Australia, Perth, Western Australia, Australia; 4 Academic Unit for Psychiatry of Old Age, St Vincent's Health, Department of Psychiatry, University of Melbourne, Melbourne, Victoria, Australia; 5 School of Psychology, Bangor University, Bangor, Wales, UK

## Abstract

**Background:**

Research evidence from observational studies suggests that cognitive activity reduces the risk of cognitive impairment in later life as well as the rate of cognitive decline of people with dementia. The Promoting Healthy Ageing with Cognitive Exercise (PACE) study has been designed to determine whether a cognitive activity intervention decreases the rate of cognitive decline amongst older adults with mild cognitive impairment (MCI).

**Methods/Design:**

The study will recruit 160 community-dwelling men and women aged 65 years of age or over with mild cognitive impairment (MCI). Participants will be randomly allocated to two treatment groups: non-specific education and cognitive activity. The intervention will consist of ten 90-minute sessions delivered twice per week over a period of five weeks. The primary outcome measure of the study is the change from baseline in the total score on the Cambridge Cognitive Score (CAMCOG). Secondary outcomes of interest include changes in memory, attention, executive functions, mood and quality of life. Primary endpoints will be collected 12, 52 and 104 weeks after the baseline assessment.

**Discussion:**

The proposed project will produce the best available evidence on the merits of increased cognitive activity as a strategy to prevent cognitive decline among older adults with MCI. We anticipate that the results of this study will have implications for the development of evidence-based preventive strategies to reduce the rate of cognitive decline amongst older people at risk of dementia.

**Trial registration:**

ACTRN12608000556347

## Background

The World's population is ageing rapidly and so is the frequency of age-related disorders. Dementia is one of the most frequent mental health disorders of older people and a leading cause of years of life lost due to disability [1]. Mild Cognitive Impairment (MCI) in old age is considered an important clinical state potentially predictive of future cognitive decline. The term MCI has been used to describe a heterogeneous group of older adults, who, whilst not fulfilling diagnostic criteria for dementia, demonstrate cognitive abilities at a level below what is considered normal for their age. Cognitive deficits may be apparent across unitary or multiple domains though the person continues to maintain a high level of functional independence [2]. Whilst this diagnostic group is at increased risk for conversion to dementia, a substantial number of individuals remain stable or even return to normal levels of functioning with time [3].

There is increasing evidence that the onset of dementia can be delayed by targeting relevant risk factors. In older adults, frequent participation in mentally stimulating leisure activities has been associated with stronger cognitive abilities (such as memory) and reduced risk of dementia [4,5]. The idea that mentally stimulating activity can influence cognitive processes is akin to the "use it or lose it" adage [6]. Few randomised controlled studies have explored this notion and critical evaluation of the research on this topic reveals a number of methodological limitations. These include the specific nature of some of the participant cohorts, the tests used to measure cognitive functioning, and the complexity associated with identifying and defining a broad range of leisure activities with regard to type and degree of cognitive stimulation (see [6] for a review).

Cognition-focused interventions (cognitive training, cognitive rehabilitation and cognitive stimulation) attempt to make use of intact domains to help maintain cognition and prevent/delay decline. These techniques have also been employed to increase functional independence and reduce caregiver burden (see [7] for a review).

Similar to mentally stimulating leisure activities, cognitive stimulation (CS) is a type of intervention that emphasises the benefits of group activities focussing on education, discussion and debate, and problem solving. In contrast, cognitive rehabilitation (CR) can be tailored towards an individual's needs, with consideration given to specific areas of impairment and an emphasis on improving everyday functioning. Another commonly used strategy is cognitive training (CT), which aims to provide the individual with a set of standardised tasks that they repeatedly perform. CT using specifically designed computer programs has become a burgeoning business and is generating increasing scientific interest.

Collectively, the research in this area has revealed that older adults (with and without dementia) show modest gains across areas related to the target intervention, and improvements on subjective measures assessing aspects of mood and quality of life have also been observed [8]. However, there is a paucity of randomised controlled trials in this area, limiting conclusions that can be drawn from existing data.

We have designed a single-blind, randomised controlled trial of an intervention that draws on methods of cognition-focussed approaches, selected with regard to suitability for older adults with MCI, and combined into a package considered interesting, engaging and acceptable to this group. The primary focus of this research is to determine whether a structured program of cognitive activity (CA) can decrease the rate of cognitive decline amongst older adults with MCI over 24 months. We hypothesize that participants allocated to the CA group will experience less cognitive decline than older adults randomised to a control education intervention.

## Methods/Design

### Background

The Promoting Healthy Ageing with Cognitive Exercise (PACE) study is a randomised controlled trial that commenced recruitment of participants in March 2007.

In late 2006, eight individuals with MCI, aged 60 years and over, were invited to offer their opinions regarding the development of a program of cognitive activity (CA), specifically designed for older adults with cognitive decline. The group confirmed the merit of such research and provided feedback with respect to the elements needed to be incorporated into the designed intervention. The general consensus was that any CA program needed to be challenging, personally stimulating and to have a social component.

In February 2007, the CA and educational interventions forming the basis of the PACE study were piloted with 10 participants (five allocated to each intervention), aged 65 and over with MCI. These individuals attended on a daily basis over a two week period and were asked to provide written feedback regarding the session material. The proposed interventions were well received, with positive feedback regarding the relevance of the content/material, format and delivery style. The education group described the program as "useful" and "informative" and the CA group, "encouraging" and "practically helpful". After collating the feedback, additional, minor modifications were made to the study protocol and recruitment began.

### Participants

#### Recruitment of older persons

Participants were community dwelling volunteers recruited from various sources such as memory clinics, local media and other ongoing research studies. Potential participants were initially screened with a semi-structured interview over the telephone and invited to visit the Western Australia Centre for Health and Ageing (WACHA), Royal Perth Hospital (RPH) for a more detailed screening assessment (clinical screen) and to provide written informed consent. The Ethics Committee of the RPH has approved the study protocol and procedures.

#### Inclusion and Exclusion Criteria

The most important defining feature of participants included in the PACE study was a diagnosis of MCI, which was ascertained at a screening assessment at the WACHA (discussed in further detail below). Individuals needed to be aged 65 years or over at their last birthday and be willing and able to travel to the WACHA. All were proficient in spoken and written English. Individuals with a diagnosis of dementia according to ICD-10 criteria for Research [9] or suffering notable cognitive impairment, as evidenced by a Mini Mental State Examination (MMSE; [10]) score of 23 or less, were excluded from the study. Additional exclusion criteria included current psychiatric disorder (e.g. depressive episode), and current history of hazardous or harmful alcohol consumption (based on the Australian Alcohol Guidelines endorsed by the National Health and Medical Research Council 2001). Those individuals with a current medical condition that prevented participation in the study tasks (such as sensory impairment) or was associated with reduced survival over a 12 month period (e.g. advanced cancer) were excluded. We also excluded from the trial people who reported a clinical history of stroke associated with permanent disability.

#### Telephone Interview

Volunteers were initially screened via a telephone interview to ascertain individuals' concerns regarding their memory. Those indicating that they had received a diagnosis of dementia were immediately excluded. The remainder were then asked about general health - both past and current concerns - as well as education and English literacy skills. Details regarding current alcohol consumption were also collected and all potential participants completed the PHQ-9 (see below) and the TICS-M (see below). Telephone interviews took approximately 10 to 30 minutes to complete depending on the extent of information needed to address inclusion and exclusion criteria. Those meeting provisional criteria for inclusion were invited to a face-to-face assessment at the WACHA to confirm that they fulfilled the study criteria.

##### Patient Health Questionnaire - Nine Item (PHQ-9) [11]

The PHQ-9 is the depression module taken from the full PHQ [12], an instrument used to make criteria-based diagnoses of depressive and other mental disorders according to the DSM-IV. In this study it was used to initially exclude individuals with depression and then to monitor the presence of depressive symptoms at follow up. Scores range from 0 to 27, with scores of 15 or greater indicative of clinically significant depression. Volunteers with a score of 15 or more on the PHQ-9 were excluded from further participation in the study.

##### The modified Telephone Interview for Cognitive Status (TICS-M)_[13]

The TICS-M was developed as a dementia screen and contains 21 items, with a total score out of 50. It takes five to ten minutes to complete and has been used to recruit for clinical trials by screening for amnestic MCI. In this study, individuals who obtained scores between 19 or greater and 38 or less were considered as possible candidates with MCI and invited to complete the next phase of recruitment. Older adults with scores lower than 19 were excluded, with this score suggesting the presence of clinically significant cognitive impairment. Those individuals with scores above the cut-off were also excluded as their performance suggested relatively intact cognitive functioning.

#### Clinic Screen

Three-hundred and twenty-four older adults completed the face-to-face assessment to establish the diagnosis of MCI according to the following criteria [2]

• Cognitive complaints and reports of decline from the individual

• Cognitive disorder as evidenced by clinical evaluation (impairment in memory and/or in another cognitive domain)

• Absence of major repercussions on daily life (the individual may however report difficulties concerning complex day to day activities)

• Absence of dementia

Cognitive disorder was established by identifying performances 1.5 standard deviations below the age and sex norms on any Consortium to Establish a Registry for Alzheimer's Disease (CERAD) cognitive task [14]. The CERAD is composed of sub-tests assessing language, memory and praxis and is considered a valid and reliable measure of cognitive function, as well as MCI and Alzheimer's disease [15]. Embedded within this test battery is the MMSE. This brief cognitive test is a commonly used screening instrument that produces a total score that can range from 0 to 30. Scores lower than 24 are reliably associated with the diagnosis of dementia or other organic mental disorders. The present study also used the MMSE to exclude participants with more severe cognitive impairment than MCI.

In addition to the cognitive screen, participants were required to score less than 16 on the World Health Organisation's Alcohol Use Disorders Identification Test (AUDIT). This self report questionnaire is considered a reliable screening tool sensitive to the detection of risky, hazardous or harmful drinking [16]. There are 10 items and supplementary questions, with questions scored on a scale of 0 to 4. Scores of 16 or above suggest "high-risk" or "harmful level" of drinking behaviour.

A self-reported medical history questionnaire was also completed by the participants and details collected regarding their medication use. A screen of participants' ability to manage complex activities of daily living was conducted using a modified version of the Structured Assessment of Independent Living Skills (SAILS [17]). Information regarding the participant's motor skills (fine and gross), language abilities and capacity to carry out tasks similar to day to day activities (e.g. money related skills, following a recipe, reading a calendar) were collected. A score of 150 represented a perfect score and those individuals with scores of less than 141, suggestive of difficulties carrying out instrumental activities of daily living, were excluded.

The screening assessment at the WACHA took approximately 30 to 40 minutes to complete. Any pertinent clinically information was reported to the relevant treating physician with the consent of the study participant. Of those who were screened, 171 met criteria and 160 completed written informed consent to participate.

### Outcome Measures and Assessment Procedures

#### Baseline Assessment

Baseline assessments were completed within three months of inclusion in the study, approximately two weeks prior to the first intervention session and prior to randomisation. Baseline and post intervention assessments took between 60 to 90 minutes to complete (including the provision of short breaks), with a two hour session allocated for the more detailed 12 and 24 month follow ups. The assessments comprised a series of tests and questionnaires, and included the following:

#### Primary outcome measure

Cambridge Cognitive Screen (CAMCOG): This is the brief neuropsychological battery of the Cambridge Examination for Mental Disorders of the Elderly - Revised (CAMDEX-R) [18] that includes a range of objective cognitive tests. It provides sub-scale scores for a number of cognitive domains as well as a global score out of 105. It takes approximately 30 minutes to administer and is very effective at differentiating between people with and without dementia [19]. There is also evidence that CAMCOG scores are sensitive to change over time [20]. The CAMCOG is the primary outcome measure of this study.

#### Secondary Outcome Measures

California Verbal Learning Test-Second Edition (Standard and Alternate Forms) (CVLT-II) [21]: This is a 16-item word list task that measures verbal learning and memory. It yields scores on immediate and delayed recall as well as recognition. Provision of standard and alternate forms minimises practice effects and are being used to monitor change in memory functions. The CVLT-II was chosen as a secondary outcome measure because low scores on word list learning tasks are associated with progression to dementia [22].

The Symbol Search sub-test from the Wechsler Adult Intelligence Scale-Third Edition (WAIS-III) [23] yields a measure of perceptual processing speed. Participants are asked to indicate whether target symbols appear in a search group of symbols. There are 60 items consisting of paired groups of symbols and the individual has 120 seconds to complete as many items as possible. Prior research has found that CA is associated with lower rates of decline on measures assessing processing ability such as perceptual speed and working memory [24], supporting the use of Symbol Search and Digit Span (see below) as an additional secondary outcome of interest.

The Digit Span sub-test from the WAIS-III [23] comprises forward and backward span components assessing individuals' auditory immediate attention and working memory. The examinee listens to a series of digits given orally by the examiner and then repeats the digits either in a forward or reverse sequence. Testing is discontinued after failure on the two trials of any series.

The Trail Making Test (TMT) [25] has two parts and measures complex visual scanning, motor speed and mental agility. In Part A the participant draws lines to connect consecutively numbered circles. Part B requires the participant to connect a sequence of numbered and lettered circles, alternating between the two sequences. The tests are timed, with participants instructed to complete the sequencing as quickly as possible.

The Controlled Word Association Test (COWAT) [25] consists of three word naming trials, with the letters FAS employed. Participants are asked to say as many words as they can think of that begin with the given letter of the alphabet, excluding proper nouns, numbers and the same word with a different suffix. A minute is given for each letter trial and performance is used as an indicator of executive functioning.

PHQ-9: We used the PHQ-9 total score, as previously described, to monitor changes in mood throughout the trial.

Leisure Activity and Frequency Questionnaire [4]: This seven-item questionnaire assesses the frequency of participation in a variety of mentally stimulating leisure activities. Given the relationship identified between leisure activity and cognitive decline [24], the aforementioned questionnaire was chosen to identify the effects that the individual's level of activity over the course of the study may have on their rate of cognitive decline.

Participants also rated their level of engagement in *physical activity *[26]. They were asked to indicate how much time they spent, over a week, taking part in vigorous and non-vigorous activity. An additional questionnaire was also added to identify the nature and quality of the participants' *social relationships *[27].

The Memory Functioning Questionnaire (MFQ) [28]: The MFQ is a 64-item questionnaire evaluating self-perception of everyday memory functioning. It was used at baseline and across follow-up assessments to determine the influence of the intervention on perceived level of memory ability.

The Quality of Life in Alzheimer's Disease (QoL-AD) [29]: This is a 13-item questionnaire completed by the participant assessing their perception of their quality of life across a number of different domains.

Participants were also asked to provide specific details regarding their educational and occupational background.

The assessment battery described above was repeated immediately after the intervention (please see details below) and again after 12 and 24 months (please see table 1). The 12 and 24 month follow-up assessments were undertaken relative to the baseline testing and also included the additional measures administered at the "Clinic Screen".

**Table 1:** Outline of the assessments and timelines of the PACE trial.

*Assessment Tool*	Telephone Screen	Clinical Screen	Baseline (0 weeks)	Post-Intervention (12 weeks)	12 months (52 weeks)	24 months (104 weeks)
TICS-M	X					

PHQ-9	X	X	X	X	X	X

CERAD		X			X	X

MMSE		X			X	X

AUDIT		X			X	X

SAILS		X			X	X

MHQ		X			X	X

CAMCOG			X	X	X	X

CVLT-II			X	X	X	X

Digit Span			X	X	X	X

Symbol Search			X	X	X	X

TMT			X	X	X	X

COWAT			X	X	X	X

LAQ			X	X	X	X

PAQ			X	X	X	X

SNSQ			X	X	X	X

MFQ			X	X	X	X

QOL-AD			X	X	X	X

The X indicates at which point of the trial the respective assessments took place. Follow-up times relate to baseline testing.

A booster telephone call took place at 24 weeks, with a face to face booster at 52 weeks.

TICS-M = The Modified Telephone Interview for Cognitive Status; PHQ-9 = Patient Health Questionnaire - Nine Item; CERAD = Consortium to Establish a Registry for Alzheimer's Disease; MMSE = Mini Mental State Examination; AUDIT = Alcohol Use Disorders Identification Test; SAILS = Structured Assessment of Independent Living Skills; MHQ = Medical Health Questionnaire; CAMCOG = Cambridge Cognitive Examination; CVLT-II = California Verbal Learning Test - Second Edition; TMT = Trail Making Tests; COWAT = Controlled Oral Word Association Test; LAQ = Leisure Activity Questionnaire; PAQ = Physical Activity Questionnaire; SNSQ = Social Network Satisfaction Questionnaire; MFQ = Memory Functioning Questionnaire; QOL-AD = Quality of Life in Alzheimer's Disease.

#### Biological Sample and DNA Collection

We also asked participants to donate a blood or saliva sample to determine the influence of common biochemical (e.g., high plasma homocysteine) and genetic factors (e.g., apolipoprotein E4 genotype) on participants' response to the intervention. The samples are being collected and processed by the Department of Clinical Pathology and Biochemistry at the RPH where they are currently stored at -80°C. All material has been batched and will only be processed at the end of the trial.

### Intervention

The interventions (both CA and education) consisted of a five-week group activity run by a qualified Neuropsychologist at the WACHA. The CA and control education groups were exposed to the same length of intervention, social interaction and contact with the program coordinator. Both the CA and educational intervention were manualised and delivered in a structured way, and all sessions (220) were audio-taped for subsequent fidelity assessment. Forty four of these sessions were randomly selected and transcribed. An independent rater used a specially devised scale to evaluate session content for consistency of concepts and issues raised across sessions according to a pre-defined criteria.

Research assistants (RAs) blinded to group allocation conducted all assessments. RAs were provided with strict instructions to avoid any potential opportunity for disclosure regarding intervention participation. The intention is that RAs undertaking data collection will be asked to guess the group membership of participants at the end of the study. This will be done to determine the effectiveness of the blinding procedures that were put in place for this project. A brief summary of each intervention is provided below.

#### CA Group

Each group consisted of 6-9 participants who took part in 90-minute sessions twice a week for five weeks (10 sessions in total). Session One introduced the nature of the program and developed familiarity within the group, with personal introductions and sharing of background information/experiences. Sessions Two and Three focussed on the cognitive domains of attention, processing speed and executive functions, how these domains change as people age and the influence they have on memory abilities. Participants were advised about strategies to manage cognitive decline associated with these domains, including a number of practical exercises. Sessions Four to Seven primarily focused on memory, with the aim of defining the processes involved in learning and retaining new information. These sessions provided participants with strategies and techniques to manage memory dysfunction. There was regular opportunity for supervised practice of such techniques in all sessions. Session Eight reviewed age associated language difficulties and aimed to provide participants with ways to manage word finding difficulties, as well as affording opportunity for undertaking language-based exercise activities. Sessions Nine to Ten were used to practice and review the previously presented material and recommended strategies, and to discuss any difficulties managing the strategies/techniques previously presented.

As part of the intervention, all participants received a folder containing the slides of each session, and copies of all completed activities.

#### Education Group

Each educational group consisted of 6 to 9 participants who took part in 90-minute sessions twice a week for five weeks (10 sessions in total). Session One followed the same format described for the CA group. Session Two covered the topic of memory functioning and dementia, aiming to provide a broad overview of how these issues can affect older people. Session Three reviewed the health benefits associated with physical activity and ways to incorporate physical activity into daily lifestyle. Sessions Four and Five were devoted to defining stress and depression as well as reviewing the cause, effects and management of these conditions. Session Six outlined changes in sleep associated with ageing and the management of sleep disturbance. In sessions Seven to Nine, focus was placed on issues of retirement including expectations, lifestyle changes, volunteer activities, cultural/societal implications of ageing and travelling. Session Ten reviewed the content of material covered in the previous weeks and provided opportunity for discussion of any questions/issues arising from the presented topics. In contrast to the CA group, there was no emphasis on skill development or the promotion of mentally stimulating activities and sessions had a greater level of mental passivity. However, as in the CA group, there was opportunity for group discussion and interaction and participants were provided with a folder containing the slides of each session.

#### Booster Sessions

All participants received a fifteen-minute "booster" telephone call six months after the baseline assessment to review and discuss the topics presented at the group sessions. Thirty minutes of additional exercise material was posted to participants in the CA group for completion prior to the booster call and served as the basis for discussion during the telephone contact. After the 12-month assessment a face-to-face group 1-hour booster session was offered to both intervention groups. This session was used to review the practical aspects of the CA and educational programs.

### Randomisation

After the baseline assessment, participants were randomly allocated to either the CA or control education interventions according to a random list of numbers generated by computer. Randomisation was undertaken in random blocks of 12 to 18, with six to nine individuals allocated to each group. The allocation list was handled by an independent investigator (OPA) who had no contact with study participants and was not involved in the supervision of staff responsible for the collection of data. The allocation table was then passed on to the investigator running the intervention (MV), who invited eligible participants to join the relevant groups. Research assistants undertaking the follow-up assessments remained blinded to group allocation.

### Sample Size and Power Calculation

At present there is no reliable data for calculating the sample size of the proposed trial. Currently available data suggests that older people living in the community lose 1.6 points per year on the CAMCOG [30]. Factoring in a possible 20% loss to follow up, with 64 people in each group we will have 80% power to detect a between-groups difference of 1.5 points on the CAMCOG. This assumes a decline that is twice as large in the educational compared with the cognitive intervention group, and although statistically this may be associated with moderate effect size (0.5), it is the minimum difference that one would consider clinically significant.

### Analysis of the Data

Changes in the CAMCOG score from baseline are the primary outcome of interest in the study. We will model these changes at 3 time points: 12 weeks (immediately after the intervention comes to an end), 52 and 104 weeks. We will use mixed effects models to analyse the data. This approach will enable us to take into account the cognitive performance of participants at baseline, as well as the intra-person correlation generated from repeated measures. Intention-to-treat analyses will be based on the use of imputation by chain equations (ICE), which will precede the use of the mixed-effects model.

We accept that participation in the cognitively non-specific educational intervention is likely to have an effect on the performance of participants. However, to ensure continued participation of the control group we deemed it crucial to provide some form of intervention or potentially face differential drop out, with the control group participants withdrawing because of lack of engagement in the study. We will model changes over time for both groups and this will enable us to determine the effect of both interventions (as well as the sustainability of this effect over 24 months) on cognitive performance.

### Comment

Our group has previously demonstrated that it is possible to delay cognitive decline in people with MCI by means of a physical activity program [31]. Observational data and basic research suggest that cognitive activity is also associated with decreased risk of cognitive impairment, though there is limited empirical evidence from randomised trials that cognitive activity can delay the progression of cognitive decline in people with MCI who are at increased risk of developing dementia. This trial has been designed according to CONSORT guidelines and has been structured to enable its reproduction in both research and clinical settings. We expect to collect the final endpoint by December 2010 and anticipate that the results of this study will have implications for the development of evidence-based preventive strategies to reduce the rate of cognitive decline amongst older people at risk of dementia.

## Competing interests

The authors declare that they have no competing interests.

## Authors' contributions

All authors are members of the PACE project group and participated in the conceptualisation and implementation of the study. MV acts as guarantor of the data and has been responsible for the day-to-day supervision of research staff and delivery of the interventions. All investigators have contributed to design the study and to obtain funding. MV and OA have drafted this manuscript, which has been critically reviewed by NL, LC and LF. All authors have approved the submission of the present paper to Trials.
